# Half-dose may be equivalent to full-dose anticoagulants in preventing venous thromboembolism after primary total joint arthroplasty: a single center comparative retrospective cohort study

**DOI:** 10.3389/fphar.2025.1680985

**Published:** 2025-10-09

**Authors:** Yinghao Wang, Zhixin Liao, Zongke Zhou, Haoyang Wang

**Affiliations:** ^1^ Department of Orthopedics and Research Institute of Orthopedics, West China Hospital, Sichuan University, Chengdu, China; ^2^ Department of Orthopedics, West China Hospital, Sichuan University, Chengdu, China

**Keywords:** total joint arthroplasty, venous thromboembolism, anticoagulant, total blood loss, reduce dose

## Abstract

**Background:**

The optimal anticoagulant administration for deep vein thrombosis (DVT) prevention following total joint arthroplasty (TJA) remains controversial. We aimed to compare the effectiveness and safeness of half-dose and full-dose anticoagulant regimen in preventing DVT following TJA surgery.

**Methods:**

This study was designed as a retrospective comparative analysis. All eligible patients received DVT prophylaxis within 2 weeks postoperatively, consisting of enoxaparin (first 2 days) and rivaroxaban (subsequent 12 days). According to the dosage of anticoagulant, patients were divided into a half-dose group (0.2 mL enoxaparin sodium and 5 mg rivaroxaban) and a full-dose DVT prophylaxis group (0.4 mL enoxaparin sodium and 10 mg rivaroxaban). The occurrence of DVT and pulmonary thromboembolism (PE) within 2 weeks postoperative, total blood loss (TBL) during the use of anticoagulants. WOMAC scores recorded at 12 months postoperative were compared between the two groups.

**Results:**

A total of 1886 patients were enrolled in this study, including 1,246 in the half-dose group and 640 in the full-dose group. No cases of PE were reported in the two groups. The DVT was detected in 11 patients (0.9%) in the half-dose group, which was not significantly different from 8 patients (1.3%) in the full-dose group, p = 0.648. Also, the WOMAC scores were comparable between the half-dose group (11.11 ± 18.01) and the full-dose group (8.88 ± 15.33), p = 0.393. Contrastively, the TBL (560.88 ± 307.69 mL) and average hospital stay (4.73 ± 1.50 days) were significantly lower in the half-dose group than that in the full-dose group (653.95 ± 333.70 mL, 5.74 ± 2.36 days), p < 0.001.

**Conclusion:**

Half-dose of DVT prophylaxis drugs during the perioperative period of TJA does not elevate DVT incidence. Furthermore, it significantly decreases TBL and shortens hospital stays, while having no adverse impact on postoperative joint function recovery. These findings may support the implementation of half-dose protocol anticoagulants in the TJA protocol.

## Introduction

Deep vein thrombosis (DVT) remains one of the substantial concerns for lower-extremity surgery, including total joint arthroplasty (TJA). Historically, inadequate preventive measures and limitations in prophylactic drugs resulted in DVT incidences exceeding 50% of TJA cohort, often leading to severe complications such as PE and mortality (Heit, 2008). Since the 1980s, various anticoagulant, such as low molecular weight heparin and factor Xa inhibitors, were recommended to reduce the incidence of symptomatic DVT to 5%–10% (Januel et al., 2012; Falck-Ytter et al., 2012; Lieberman and Bell, 2021), marking a significant advancement in DVT prevention for TJA.

However, the usage of anticoagulants was associated with various concerns. Hidden blood loss after TJA can range from 1,000 to 1,800 mL (Liu et al., 2011; Kalairajah et al., 2005; Ma et al., 2014; Hu et al., 2018). Under the influence of anticoagulant drugs, additional bleeding may come unasked postoperatively, which likely resulting in complications like swelling, hematoma formation, and increased risk of wound healing issues and prosthetic joint infections, and even gastrointestinal bleeding and intracranial hemorrhage (Parvizi et al., 2007).

Multiple combined measures, along with postoperative anticoagulant administration, have markedly decreased DVT incidence following TJA (Xie et al., 2015; Gesell et al., 2013; Lieberman and Heckmann, 2017; Westrich et al., 2006). Under these conditions, adjusted-dose of anticoagulant have been attempted in recent datum to seek a balance between thrombosis prevention and bleeding avoidance (Faour et al., 2019; Merkow et al., 2021; Faour et al., 2018), nevertheless, the increased risk of bleeding remains not well-balanced in these existing anticoagulant regimens.

Therefore, our joint surgery center, which handles over 3000 TJA cases annually, conducted this single-center, retrospective study to establish an optimal anticoagulant regimen for balancing thrombosis prevention and bleeding avoidance by comparing the effectiveness and safeness of full-dose and half-dose anticoagulant regimens in TJA protocol.

## Methods

### Ethical review statement

On 19 August 2024, the Biomedical Ethics Committee of our hospital approved this study (Ethics Committee Number Trial No. 1621, 2024). And it was registered in the Chinese Clinical Trial Registry, the Unique Identifying Number of this study is ChiCTR2400091009.

### Patients selection

This was a retrospective study approved by the institutional review board in our hospital with written informed consent obtained. A total of 2,511 patients who underwent TJA due to primary unilateral hip or knee joint diseases unresponsive to conservative treatment from 2018 to 2023 were identified. Patients with history of DVT, calf muscle vein thrombosis, superficial vein thrombosis, and pulmonary embolism (PE), abnormal coagulation function or long-term use of anticoagulant drugs, renal insufficiency, extremely low or high body weight, and allergic to related medications were excluded.

### Perioperative management

Following the standard surgical protocol, a medial patellar joint approach was employed for total knee arthroplasty (TKA) and a posterior lateral approach was employed for total hip arthroplasty (THA) under general anesthesia. Patients undergoing TKA were equipped with pneumatic tourniquet starting from the initial skin incision and continuing until wound closure. Patients undergoing TKA used posterior-stable cemented prosthesis (DePuy Synthes), and patients undergoing THA used bio-type total hip prosthesis (DePuy Synthes). Tranexamic acid (1 g) was administered intravenously 10 min before skin incision, again 30 min prior to wound closure, and 3 h, 6 h and 12 h post-surgery. No drains were placed for all patients.

All patients received standardized mechanical thromboprophylaxis in accordance with current clinical guidelines, comprising intermittent pneumatic compression (IPC) devices, graduated compression stockings (GCS), ankle pump exercises and protocol-driven early ambulation.

Patients in full-dose group received subcutaneous injection of enoxaparin sodium of 0.4 mL (4,000 IU) (Clexane, Sanofi) once every 24-h starting from 8 h post-surgery and extending for 48 h, and followed by orally rivaroxaban of 10 mg (Xarelto, Bayer) once daily starting from 3 days post-surgery and extending for 14 days. Contrastively, patients in half-dose group received enoxaparin sodium of 0.2 mL (2,000 IU) and rivaroxaban of 5 mg in the same manner.

### Data collection

Patient’s demographic variables, including age, gender, body mass index, medical history, laboratory coagulation indicators were collected. The primary outcome measures included the occurrence of DVT of lower limb vein above popliteal vein detected by Color Doppler ultrasound examination at the 14-days follow-up, and the TBL which was calculated according to the hematocrit difference between 8 h post-surgery and 14 days post-surgery using the formula described by Gross and Nadler (Gross, 1983; Nadler et al., 1962). The secondary outcome measures included the occurrence of PE within 14 days postoperative, the length of hospitalization, the incidence of postoperative bleeding events within 14-day post-surgery, and WOMAC score obtained at 12 months post-surgery to assess the pain, stiffness, and functionality of the operated joint.

### Statistical analysis

Continuous data was expressed as mean and standard deviation (SD) or median with interquartile range if not normally distributed. The independent samples t-test was used for normally distributed data, while the Mann-Whitney U test was employed for skewed data. Categorical data were expressed as numbers and percentages, analyzed using the Pearson chi-square test. Statistical analyses were conducted using SPSS (version 23; IBM), with significance set at p < 0.05.

## Results

### Baseline characteristics

A total of 625 patients were excluded from this study. Our final analysis encompassed 1,886 patients, including 1,246 patients in half-dose group and 640 patients in full-dose group, to compare the effectiveness and safeness of the two regimens anticoagulant. There were 418 males and 828 females, with a mean age of 55.80 ± 12.72 years in half-dose group, and 220 males and 420 females, with a mean age of 54.77 ± 15.83 years in full-dose group. The two groups presented with a mean BMI of 24.96 ± 3.55 and 24.76 ± 3.92, respectively. There were no significant differences in demographic between the half-dose group and the full-dose group (Tables 1, 2).

**TABLE 1:** Population characteristics.

Characteristic	Half dose (N = 1,246)	Full dose (N = 640)	P value
Age (years)	55.80 ± 12.72	54.77 ± 15.83	**0.428**
Height (cm)	158.75 ± 8.31	158.54 ± 9.15	**0.777**
Weight (kg)	63.01 ± 10.69	62.09 ± 10.30	**0.305**
BMI* (kg/m^2^)	24.96 ± 3.55	24.76 ± 3.92	**0.504**
Female sex (no. [%] of patients)	828 (66.5)	420 (65.6)	**0.835**
Risk factors (no. [%] of patients)			
Hypertension	342 (27.5)	160 (25.0)	**0.512**
Diabetes	100 (8.0)	48 (7.5)	**0.817**
Coronary heart disease	15 (1.2)	8 (1.3)	**0.960**
Smoking	137 (11.0)	72 (11.3)	**0.923**
Alcohol intake	159 (12.8)	88 (13.8)	**0.617**
Preop. laboratory values			
PT† (sec)	10.50 ± 0.64	10.42 ± 0.68	**0.151**
INR‡	0.95 ± 0.06	0.94 ± 0.06	**0.086**
APTT§ (sec)	26.69 ± 3.41	27.14 ± 2.78	**0.110**
D-dimer (mg/L)	0.91 ± 3.18	1.09 ± 2.87	**0.510**
FDP∥ (mg/L)	1.01 ± 3.02	1.6 ± 6.42	**0.254**
Platelet count (×109/L)	196.16 ± 65.01	193.92 ± 73.55	**0.713**
Hemoglobin (g/L)	135.78 ± 14.25	135.43 ± 16.83	**0.798**
Hct¶ (L/L)	0.42 ± 0.04	0.42 ± 0.05	**0.726**
Predicted blood volume (mL)	2,832.93 ± 370.90	2,801.79 ± 354.71	**0.315**

* Body mass index, † Prothrombin time, ‡ International normalized ratio, § activated partial thromboplastin time, ∥ fibrinogen degradation product, and ¶ hematocrit.

**TABLE 2:** Population characteristics according to surgical subgroup.

Characteristic	Total knee arthroplasty (N = 887)		P value	Total hip arthroplasty (N = 999)		P value
	Half dose (N = 543)	Full dose (N = 344)		Half dose (N = 703)	Full dose (N = 296)	
Age (years)	61.85 ± 9.88	59.37 ± 15.34	**0.151**	51.14 ± 12.71	49.42 ± 14.75	**0.280**
Height (cm)	157.19 ± 7.75	157.03 ± 8.80	**0.863**	159.96 ± 8.53	160.28 ± 9.30	**0.756**
Weight (kg)	64.68 ± 10.14	63.34 ± 9.99	**0.253**	61.72 ± 10.93	60.64 ± 10.53	**0.419**
BMI (kg/m^2^)	26.16 ± 3.61	25.80 ± 4.21	**0.394**	24.03 ± 3.22	23.55 ± 3.17	**0.222**
Female sex (no. [%] of patients)	424 (78.1)	240 (69.8)	**0.089**	404 (57.5)	180 (60.8)	**0.580**
Risk factors (no. [%] of patients)						
Hypertension	212 (39.0)	124 (36.0)	**0.596**	130 (18.5)	36 (12.2)	**0.177**
Diabetes	73 (13.4)	32 (9.3)	**0.287**	27 (3.8)	16 (5.4)	**0.513**
Coronary heart disease	15 (2.8)	4 (1.2)	**0.381**	0	4 (1.4)	**0.002**
Smoking	55 (10.1)	32 (9.3)	**0.812**	82 (11.7)	40 (13.5)	**0.640**
Alcohol intake	67 (12.3)	56 (16.3)	**0.311**	87 (12.4)	32 (10.8)	**0.696**
Preop. laboratory values						
PT (sec)	10.48 ± 0.64	10.25 ± 0.65	**0.003**	10.51 ± 0.64	10.61 ± 0.66	**0.179**
INR	0.95 ± 0.06	0.94 ± 0.06	**0.361**	0.95 ± 0.06	0.94 ± 0.06	**0.145**
APTT (sec)	26.49 ± 3.06	26.96 ± 2.95	**0.170**	26.85 ± 3.66	27.35 ± 2.57	**0.252**
D-dimer (mg/L)	0.81 ± 1.16	1.07 ± 3.29	**0.474**	0.99 ± 4.11	1.1 ± 2.3	**0.811**
FDP (mg/L)	0.98 ± 3.32	1.25 ± 6.48	**0.551**	1.04 ± 2.77	2.01 ± 6.36	**0.197**
Platelet count (×109/L)	195.21 ± 66.98	194.38 ± 65.64	**0.915**	196.90 ± 63.49	193.38 ± 82.26	**0.722**
Hemoglobin (g/L)	134.98 ± 12.99	136.38 ± 15.11	**0.417**	136.40 ± 15.14	134.31 ± 18.68	**0.355**
Hct (L/L)	0.41 ± 0.04	0.42 ± 0.04	**0.320**	0.42 ± 0.05	0.41 ± 0.05	**0.249**
Predicted blood volume (mL)	2,882.71 ± 350.18	2,837.70 ± 339.31	**0.266**	2,794.47 ± 381.96	2,760.06 ± 369.74	**0.460**

### Outcome measures

The occurrence of DVT in the half-dose group was 11 patients, with the incidence of 0.8%, which was not significant differ to that of 8 patients (incidence of 1.3%) in the full-dose group (p = 0.648) (Table 3). When it comes to subgroup analysis differentiated by surgical procedure, the incidence of DVT in half-dose group and full-dose group was 0.7% (4/543) and 1.2% (4/344) in TKA subgroup, and 1.0% (7/703) and 1.4% (4/296) in THA subgroup, respectively. Similarly, there were no significant differences in the incidence of DVT between half-dose and full-dose regimens in either TKA or THA subgroup (p = 0.679 for TKA subgroup and p = 0.773 for THA subgroup).

**TABLE 3:** Incidence of outcomes after total hip and knee arthroplasty.

Outcome	Half dose (N = 1,246)	Full dose (N = 640)	P value
Primary outcome			
Postop. DVT (no. [%] of patients)	11 (0.8)	8 (1.3)	**0.648**
Pulmonary embolism	0	0	
Death	0	0	
Secondary outcome			
Total blood loss (mL)	560.88 ± 307.69	653.95 ± 333.70	**0.000**
Hospital stays (day)	4.73 ± 1.50	5.74 ± 2.36	**0.000**
Womac index	11.11 (14.00)	8.88 (12.00)	**0.538**
Pain	1.57 (2.00)	1.43 (2.00)	**0.774**
Rigid	0.62 (0)	0.76 (1.00)	**0.243**
Function	8.92 (12.00)	6.69 (9.00)	**0.498**
Bleeding	0	0	

The mean TBL was 560.88 ± 307.69 mL in the half-dose group, which was significantly less than that of 653.95 ± 333.70 mL in the full-dose group (p < 0.001) (Table 3). In terms of the subgroup analysis, the half-dose group also had significantly less mean TBL in the TKA and THA subgroups (513.02 ± 254.17 mL and 597.84 ± 338.94 mL) than that in the full-dose group (653.95 ± 333.70 mL and 610.18 ± 320.17 mL) (p < 0.05) (Table 4).

**TABLE 4:** Incidence of outcomes according to surgical subgroup.

Outcome	Total knee arthroplasty (N = 887)		P value	Total hip arthroplasty (N = 999)		P value
	Half dose (N = 543)	Full dose (N = 344)		Half dose (N = 703)	Full dose (N = 296)	
Primary outcome						
Postop. DVT (no. [%] of patients)	4 (0.7)	4 (1.2)	**0.679**	7 (1.0)	4 (1.4)	**0.773**
Pulmonary embolism	0	0		0	0	
Death	0	0		0	0	
Secondary outcome						
Total blood loss (mL)	513.02 ± 254.17	610.18 ± 320.17	**0.008**	597.84 ± 338.94	704.82 ± 343.96	**0.010**
Hospital stays (days)	5.16 ± 1.08	6.19 ± 2.37	**0.000**	4.40 ± 1.69	5.22 ± 2.25	**0.003**
Womac index	9.28 (11.00)	7.36 (10.00)	**0.405**	12.02 (15.00)	11.67 (15.00)	**0.479**
Pain	1.37 (2.00)	1.24 (2.00)	**0.659**	1.67 (2.00)	1.78 (3.00)	**0.750**
Rigid	0.61 (0)	0.73 (1.00)	**0.413**	0.63 (0)	0.83 (1.00)	**0.280**
Function	7.30 (9.00)	5.39 (8.00)	**0.411**	9.73 (13.00)	9.06 (12.00)	**0.460**
Bleeding	0	0		0	0	

The length of the hospital stays was 4.73 ± 1.50 days in the half-dose group, which was significantly shorter than that in the full-dose group (5.74 ± 2.36 days) (p < 0.001) (Table 3). In TKA and THA subgroup, it was also significantly shorter in the half-dose group (5.16 ± 1.08 and 4.40 ± 1.69 days) than that in the full-dose group (6.19 ± 2.37 and 5.22 ± 2.25 days) (p < 0.05) (Table 4).

At 1 year postoperatively, the WOMAC scores of half-dose group and full-dose group were 11.11 ± 18.01 and 8.88 ± 15.33, there were no significant difference between the two groups (p = 0.538). In detail, the pain scores, stiffness scores and functional limitation scores were 1.57 ± 2.92, 0.62 ± 1.59 and 8.92 ± 14.43, respectively in half-dose group, were similar to those of 1.43 ± 2.82, 0.76 ± 1.77 and 6.69 ± 11.53, respectively in full-dose group (p = 0.774, 0.243, 0.498) (Table 3). Similarly, no significant differences were observed in WOMAC scores, including the pain scores, stiffness scores and functional limitation scores between the TKA or THA subgroups (Table 4). No cases of PE or mortality were observed in both half-dose and full-dose groups.

## Discussion

The substantive characteristics of potential elevated bleeding in scenario of preventing DVT by anticoagulants usage remains a substantial and increasing concern for joint surgeons (Kalathottukaren et al., 2018; Allouchery et al., 2023; Miesbach and Seifried, 2012). An optimal anticoagulant regimen that minimizes additional bleeding without sacrificing the preventive effect of DVT is still being explored. We compared the efficacy of half-dose and full-dose anticoagulants in preventing DVT following primary TJA. Our key results indicated that, compared to the full-dose regimen, half-dose regimen did not significantly increase the incidence of DVT and had less TBL. These findings suggest that the half-dose DVT prophylaxis drug is an optimal choice for DVT prevention following TJA.

Recent studies have explored the efficacy of low-dose antithrombotic (antiplatelet) agent following primary TJA. Faour M’s study demonstrated that, in patients undergoing THA, the incidence of DVT had no significant difference between 81 mg and 325 mg aspirin group (Faour et al., 2019). Similarly, Lavu MS and other colleagues reported that low-dose (81 mg) aspirin could effectively prevents DVT following TJA (Merkow et al., 2021; Faour et al., 2018; Lavu et al., 2024; Shafiei et al., 2023). We further support these findings by showing that the combination of half-dose enoxaparin and rivaroxaban effectively prevents DVT following primary TJA, with no significant difference compared to the full-dose regimen. Currently, no studies have conclusively demonstrated whether reduced-dose enoxaparin maintains sufficient anticoagulant efficacy. Our analysis suggests that mechanical DVT prophylaxis may partially substitute the anticoagulant effects, thus, half-dose regimen combined with mechanical interventions achieves comparable DVT prophylaxis efficacy to full-dose regimen.

Minimizing perioperative blood loss in TJA constitutes an additional pivotal objective in optimizing anticoagulation dosage. The current body of research lacks sufficient evidence regarding the correlation between anticoagulant dosage regimens and their quantitative impact on blood loss. Our study demonstrates a statistically significant reduction in blood loss from 653.95 ± 333.70 mL to 560.88 ± 307.69 mL (p < 0.001) following the implementation of half-dose regimen, thereby substantiating the clinical efficacy of dose reduction in reducing blood loss. The less TBL associated with half-dose regimen contributes to shortened hospital stays, thereby facilitating enhanced postoperative recovery.

Our study has several limitations. The findings are constrained by the retrospective design and variations in surgical techniques among different surgeons. Additionally, we were unable to assess patient compliance with rivaroxaban after discharge, which may affect the accuracy and credibility of our results. Due to the inherent limitations of our retrospective study design, we were unable to systematically quantify and analyze critical postoperative outcome measures, including extremity swelling indices and the incidence/severity grading of ecchymosis, which represent important parameters in assessing anticoagulation-related complications.

## Conclusion

In conclusion, reducing the dosage of DVT prophylaxis during the perioperative period of TJA did not elevate the incidence of DVT. It also reduced the TBL, shortened the hospital stays, and contributed to postoperative joint functional recovery. These findings collectively suggest that half-dose DVT prophylaxis represents a clinically viable and potentially superior alternative for DVT prevention after TJA.

## Data Availability

The raw data supporting the conclusions of this article will be made available by the authors, without undue reservation.
